# Inflammation has synergistic effect with nicotine in periodontitis by up‐regulating the expression of α7 nAChR via phosphorylated GSK‐3β

**DOI:** 10.1111/jcmm.14986

**Published:** 2020-01-13

**Authors:** Zhifei Zhou, Fen Liu, Lulu Wang, Bin Zhu, Yujiang Chen, Yang Yu, Xiaojing Wang

**Affiliations:** ^1^ State Key Laboratory of Military Stomatology & National Clinical Research Center for Oral Diseases & Shaanxi Clinical Research Center for Oral Diseases Department of Pediatric Dentistry School of Stomatology Air Force Medical University Xi’an China; ^2^ Department of Stomatology General Hospital of Tibetan Military Command Lhasa China; ^3^ Department of Stomatology Northwest Women’s and Children’s Hospital Xi’an China; ^4^ State Key Laboratory of Military Stomatology & National Clinical Research Center for Oral Diseases & Shaanxi Engineering Research Center for Dental Materials and Advanced Manufacture Department of Periodontology School of Stomatology Air Force Medical University Xi’an China; ^5^ Stomatological Hospital of Chongqing Medical University & Chongqing Key Laboratory of Oral Diseases and Biomedical Sciences & Chongqing Municipal Key Laboratory of Oral Biomedical Engineering of Higer Education Chongqing China

**Keywords:** bone metabolism, GSK‐3β, nicotine, periodontal ligament stem cells, periodontitis, α7 nicotinic acetylcholine receptor

## Abstract

Periodontitis is the leading cause of adult tooth loss, and those who smoke are at an increased risk of developing periodontitis. α7 nicotinic acetylcholine receptor (α7 nAChR) is proposed to mediate the potential synergistic effect of nicotine and inflammation in smoking‐related periodontitis. However, this has not been experimentally demonstrated. We isolated and cultured human periodontal ligament stem cells (PDLSCs) from healthy and inflamed tissues. PDLSCs were treated with either inflammatory factors or nicotine. We measured expression of genes that are associated with osteogenic differentiation and osteoclast formation using RT‐qPCR and Western blot analyses. Besides, immunohistochemical staining, micro‐CT analysis and tartaric acid phosphatase staining were used to measure α7 nAChR expression and function. Inflammation up‐regulated α7 nAChR expression in both periodontal ligament tissues and PDLSCs. The up‐regulated α7 nAChR contributed to the synergistic effect of nicotine and inflammation, leading to a decreased capability of osteogenic differentiation and increased capability of osteoclast formation‐induction of PDLSCs. Moreover, the inflammation‐induced up‐regulation of α7 nAChR was partially dependent on the level of phosphorylated GSK‐3β. This study provides experimental evidence for the pathological development of smoking‐related periodontitis and sheds new light on developing inflammation and α7 nAChR‐targeted therapeutics to treat and prevent the disease.

## 1 INTRODUCTION

Periodontitis is a disease caused by bacterial infection.^1^ At present, it is the leading cause of adult tooth loss.^2^, ^3^ Periodontitis not only increases the risk of oral disease, but also increases the risk of systemic diseases.^4^, ^5^ Therefore, the World Health Organization has called for increased attention and proposed integrated strategies to control and prevent periodontal inflammation globally.^6^

Smoking is a significant risk factor for periodontitis.^7^, ^8^, ^9^ Nicotine is one of the most toxic substances in cigarettes.^10^, ^11^ It has been revealed that α7 nicotinic acetylcholine receptor (α7 nAChR), the major nicotine receptor, is functionally expressed both in healthy tissue–derived periodontal ligament stem cells (H‐PDLSCs) and in inflammatory tissue–derived PDLSCs (I‐PDLSCs) .^12^ More importantly, the destructive effect of nicotine on periodontal tissues is achieved mainly through its interaction with α7 nAChR.^13^, ^14^, ^15^ It has been widely accepted that smoking promotes the destructive effect of inflammation in periodontitis.^10^ However, it is also well recognized that smoking itself could not cause periodontitis independent of inflammation. Since very few studies have focused on the potential synergistic effect of nicotine and inflammation, a systematic investigation on this issue could provide a comprehensive explanation regarding the pathological mechanisms of smoking‐related periodontitis.

It has been confirmed that there might be a bidirectional regulation between α7 nAChR and certain inflammatory factors like interleukin‐1β (IL‐1β) and tumour necrosis factor‐α (TNF‐α).^16^, ^17^ Moreover, the inflammatory response of PDLSCs could activate multiple signalling pathways, such as those involving glycogen synthase kinase‐3β (GSK‐3β).^18^ Furthermore, in the central nervous system, increased expression of phosphorylated GSK‐3β can up‐regulate the expression and promote the function of α7 nAChR, thus suppressing the progression of degenerative diseases.^19^ Our previous study indicated that TNF‐α significantly increases phosphorylated GSK‐3β levels in bone marrow–derived mesenchymal stem cells and PDLSCs.^20^ However, whether and how GSK‐3β signalling is involved in the pathogenesis of smoking‐related periodontitis has not been fully elucidated.

In this study, we investigated the changes of α7 nAChR expression in periodontal tissues and PDLSCs under inflammatory conditions and further clarified if α7 nAChR and inflammation synergistically affect osteogenesis and osteoclastogenesis of PDLSCs. Our study provides the experimental basis to further investigate the mechanisms of smoking‐related periodontitis and sheds new light on developing inflammation and α7 nAChR‐targeted therapeutics to treat and prevent the disease.

## 2 MATERIALS AND METHODS

### 2.1 Study subjects and ethics statement

Patients were recruited from the Department of Maxillofacial Surgery or the Department of Periodontology at the School of Stomatology, Air Force Medical University. Due to orthodontic treatment needs, healthy premolars (n = 16) or third molars (n = 9) without caries and periodontal and periapical inflammation were extracted from eight participants ranging in age from 11 to 34 years (mean age: 19.3 years). Teeth (n = 14) that could not be reserved from patients with periodontitis were collected from five participants ranging in age from 27 to 35 years (mean age: 33.7 years). All teeth met the clinical and radiographical diagnosis of chronic periodontitis.^1^ Participants did not present with any systemic diseases and had no smoking history and no special medication history.

Ethical approval was obtained from the ethics committee of the School of Stomatology, Air Force Medical University (Committee's reference number: IRB‐REV‐2016093). All participants and guardians of the patients were informed on the objectives of this study. Written consents were obtained from all participants prior to conducting the study.

### 2.2 Isolation and culture of human PDLSCs

Periodontal tissues were gently scraped from the middle third of the roots and cut into small pieces of approximately 1 mm^3^. These pieces were digested using type I collagenase (Sigma‐Aldrich, Spruce) for 15 minutes and then resuspended in alpha minimum essential medium (α‐MEM; HyClone) supplemented with 10% (v/v) foetal bovine serum (HyClone). Periodontal ligament tissues were seeded in a six‐well plate (Corning) and cultured at 37°C in a humidified atmosphere with 95% air and 5% CO_2_. The medium was changed every 3 days. The limiting dilution technique was applied to obtain single colony‐derived cell strains for further in vitro cultivation, as previously reported.^21^

### 2.3 Colony formation assay and toluidine blue staining

To assess the colony formation efficiency, a total of 1 × 10^3^ cells at the third passage were seeded into 100‐mm dishes (Corning). After 14 days of culture, cells were fixed using 4% paraformaldehyde (Henglee) and stained using 1% toluidine blue (Hengyuan) for 20 minutes at room temperature. Aggregates of 50 or more cells were considered colonies. The colony formation efficiency was calculated by dividing the total number of colonies by the total number of seeded cells.

### 2.4 Flow cytometric analysis for surface markers

PDLSCs were labelled with antibodies against mesenchymal cell surface markers and analysed using flow cytometry, as described previously.^15^ Briefly, to immunophenotype the PDLSCs, 1 × 10^5^ cells at the third passage were incubated with phycoerythrin‐conjugated or fluorescein isothiocyanate‐conjugated mouse monoclonal antibodies against human Stro‐1 (Abcam), CD146 (eBioscience), CD105 (BioLegend), CD29 (Abcam), CD166 (BioLegend), CD31 (eBioscience), CD14 (Boster) and CD45 (eBioscience) per the manufacturers’ instructions. Antibody labelling was conducted at 4°C in the dark for 1 hour. A cell suspension without antibodies served as the blank control. The cells were subjected to flow cytometric analysis using a flow cytometer (BD FACSCalibur; BD Biosciences). Data were analysed with the BD CellQuest Pro Software (BD Biosciences), and representative results from one of three independent experiments are shown.

### 2.5 Osteogenic/adipogenic differentiation induction of hPDLSCs

H‐PDLSCs or I‐PDLSCs were seeded in six‐well plates at a concentration of 5 × 10^4^ cells/well and cultured until they reached 80% confluence. The medium was then changed to osteogenic differentiation induction medium [α‐MEM supplemented with 5% FBS, 50 mg/mL ascorbic acid (Westang), 10 nmol L^−1^ dexamethasone (Sigma‐Aldrich) and 5 mmol L^−1^ β‐glycerophosphate (Sigma‐Aldrich)] or adipogenic differentiation induction medium [α‐MEM supplemented with 5% FBS, 0.5 mmol L^−1^ methylisobutylxanthine (Sigma‐Aldrich), 1 µmol L^−1^ dexamethasone (Sigma‐Aldrich), 10 µg/mL insulin (Yeasen) and 60 mmol L^−1^ indomethacin (Sigma‐Aldrich)]. After culturing for 4 weeks, the cells were fixed using 4% paraformaldehyde and stained with a fresh solution of Alizarin Red (Klamar) or Oil Red O (Sigma‐Aldrich) for 20 minute at room temperature. The mineral nodules and lipid droplets were observed using a phase‐contrast microscope (Olympus).

### 2.6 Real‐time quantitative polymerase chain reaction (RT‐qPCR)

Total RNA was extracted using the Trizol reagent (Invitrogen) according to the manufacturer's protocol. RNA was reverse transcribed into complementary DNA (cDNA) with an RNA reverse transcription kit (Takara) following the manufacturer's protocol. The cDNA was amplified using primers (sequences and their accession number to GenBank are listed in Table S1) specific for alkaline phosphatase (*ALP*), runt‐related transcription factor 2 (*RUNX2*), bone sialoprotein (*BSP*), osteocalcin (*OCN*), receptor activator of nuclear factor‐κB ligand (*RANKL*), osteoprotegerin (*OPG*), α7 nAChR (*CHRNA7*), *GSK3B* and β‐actin (*ACTB*).

Quantitative PCR was performed in a volume of 20 μL using SYBR Premix Ex TaqTM II PCR kit (Takara). PCR conditions were selected according to the suggested protocol for the CFX Connect Real‐Time PCR Detection System (Bio‐Rad). β‐actin was used as an internal reference gene, and the 2^−ΔΔCt^ method was used to calculate expression of target genes between the experimental group and control group.

### 2.7 Western blot analysis

Western blot was performed according to a previously described protocol.^22^ The primary antibodies used in this study included rabbit anti‐human ALP (1:2000; Abcam), rabbit anti‐human RUNX2 (1:500; Santa Cruz), mouse anti‐human BSP (1:500; Santa Cruz), mouse anti‐human OCN (1:500; Santa Cruz), mouse anti‐human RANKL (1:500; Santa Cruz), rabbit anti‐human OPG (1:500; Santa Cruz), mouse anti‐human α7 nAChR (1:1000; BioLegend), mouse anti‐human GSK‐3β (1:1000; Cell Signaling), rabbit anti‐human phosphorylation GSK‐3β (1:1000; Cell Signaling) and mouse anti‐human β‐actin (1:2000; Comwin). Horseradish peroxidase‐conjugated goat anti‐rabbit and goat antimouse secondary antibodies (1:5000; Jackson) were used. β‐actin served as the internal reference, and semi‐quantitation of protein expression was conducted by determining the intensity of the targeted protein bands using densitometry and Photoshop CS6 (Adobe Systems). Representative results from one of three independent experiments are shown.

### 2.8 Immunohistochemical staining

Immunohistochemical staining was performed as previously described.^21^ Paraffin serial sections (4 μm) were obtained, and immunohistochemical evaluations were performed using the following primary antibodies: mouse anti‐human α7 nAChR (1:1000; BioLegend), rabbit anti‐human ALP (1:1000; Abcam), rabbit anti‐human RUNX2 (1:200; Santa Cruz), mouse anti‐human GSK‐3β (1:250; Cell Signaling) and mouse anti‐human phosphorylated GSK‐3β (1:50; Cell Signaling). Biotin‐labelled secondary antibodies, including goat antimouse and goat anti‐rabbit antibodies (ZSGB‐bio), were used. Measurements of integrated optical density were conducted using Image Pro Plus version 6.0 software (Azure). Representative results from one of three independent experiments are shown.

### 2.9 Lentiviral infection for gene knock‐down

hPDLSCs were seeded in a 12‐well plate (Corning). When the cells reached 50%‐70% confluence, they were infected with lentiviral particles expressing *α7 nAChR*‐specific shRNA or *GSK‐3β*‐specific shRNA (Santa Cruz) according to the manufacturer's protocol. Briefly, polybrene (Santa Cruz) was added to the α‐MEM culture medium at a final concentration of 5 μg/mL. At 24 hours after incubation with the lentiviral particles, the cells were cultured with normal α‐MEM and were routinely passaged. Puromycin dihydrochloride (Santa Cruz) at a concentration of 5 μg/mL was used to select stable clones with targeted knock‐down of *α7 nAChR* or *GSK‐3β*. Control lentiviral particles (Santa Cruz) were used to establish the negative control clones. All resistant clones were subcultured for further use. RT‐qPCR and Western blot assays were conducted to analyse gene knock‐down efficiency.

### 2.10 Animal experiments

Nude mice (6‐week‐old, males) were purchased from Vital River Laboratory Animal Technology Company and maintained in the Animal Center of Air Force Medical University. hPDLSCs with indicated treatments were implanted subcutaneously into the nude mice according to previously described protocols.^21^ Briefly, treated hPDLSCs were seeded in six‐well plates at a density of 2 × 10^5^ cells/well. Porous bioceramic hydroxyapatite (HAP) cylinders (3 mm × 5 mm; Sichuan University) were placed in the wells as the scaffold material. Osteogenic differentiation induction medium was then applied for approximately 14 days until cell sheets had formed. The cell‐HAP structures were then implanted subcutaneously into the dorsal region of nude mice. After 8 weeks, the mice were killed and the implants were harvested for micro‐computed tomography (micro‐CT) scanning or demineralized in a solution of 17% ethylene diamine tetraacetic acid (Brtchem) at 37°C for immunohistochemical staining. The animal study was approved by the Animal Care Committee of the Air Force Medical University, and all experiments involving animals adhered to the approved protocols.

### 2.11 Micro‐CT scanning

The cell‐HAP implants were analysed using a micro‐CT device (Siemens AG). Scanning was performed at a 70 kV, 114 μA condition with an isotropic resolution of 10.5 μm. Sectional images of 2240 × 2240 pixels were obtained. A 1‐mm section perpendicular to the long axis of the cylindrical scaffold material was taken in the middle, and three‐dimensional images were reconstructed. Then, the ratios of the bone volume to the total volume and trabecular spacing were quantified. Images from one representative experiment are shown, and the quantitative analyses are summarized from three independent experiments.

### 2.12 Tartaric acid phosphatase (TRAP) staining

The mouse monocyte/macrophage cell line RAW264.7 was purchased from the American Type Culture Collection (ATCC) and maintained in Dulbecco's modified eagle's medium (HyClone) supplemented with 10% FBS. Differently treated hPDLSCs were co‐cultured with RAW264.7 cells, and TRAP staining was performed as previously reported.^22^ Briefly, hPDLSCs were seeded into 24‐well plates (Corning; 1 × 10^5^ cells/mL/well). After 12 hours, RAW264.7 cells (1 × 10^6^ cells/mL/well) were directly added into the α‐MEM culture medium containing 30 ng/mL human macrophage colony stimulatory factor (Wobai). After 14 days, the cells were subjected to TRAP staining using an acid phosphatase leucocyte kit (Sigma‐Aldrich). Ten different visual fields in each group were randomly selected to calculate the number of TRAP‐positive cells.

### 2.13 hPDLSC treatment with IL‐1β/TNF‐α, nicotine and α‐bungarotoxin (α‐BTX)

H‐PDLSCs were seeded into six‐well plates at a density of 5 × 10^4^ cells/well. When cells reached 80% confluence, IL‐1β (5 ng/mL; Novoprotein, Shanghai, China) and TNF‐α (10 ng/mL; Novoprotein) were added into normal α‐MEM medium or osteogenic differentiation induction α‐MEM medium. When cells reached 80% confluence in the six‐well plates, a nicotine sulphate solution (10^−9^ mol/L; Sigma‐Aldrich) and/or α‐BTX (10^−8^ mol/L; Tocris) was added to the culture medium. Cells were used for further experiments after indicated treatment periods.

### 2.14 Statistics

All experiments were performed in triplicate with hPDLSCs from at least three different patients. All results are presented as the mean ± standard deviation. A two‐tailed unpaired Students’ *t* test analysis was used to analyse data within two groups. A one‐way analysis of variance followed by Tukey's post‐test was used to analyse data within more than two groups. All data were analysed using SPSS software version 19.0 (IBM). A *P*‐value less than .05 was considered statistically significant.

## 3 RESULTS

### 3.1 Isolation and identification of H‐PDLSCs and I‐PDLSCs

**Figure 1:**
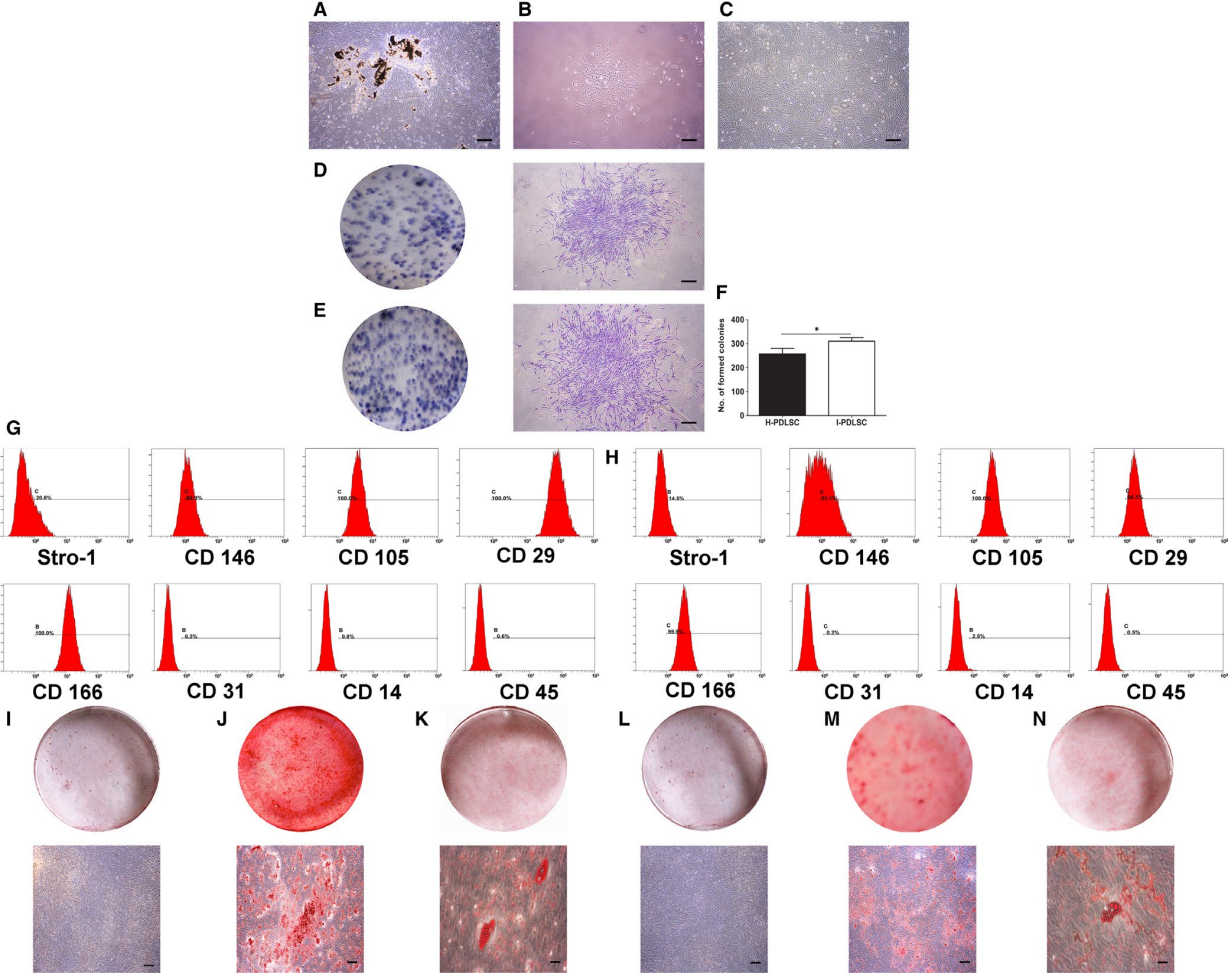
Isolation, culture, surface marker characterization and differentiation of hPDLSCs. A, Representative image of primary cultured periodontal ligament cells derived from periodontal ligament tissues; B, C, Representative images of initially selected single‐cell clones of PDLSCs (B), and PDLSCs at 14 d after further culturing (C); D, E, Representative images of toluidine blue staining show clone formations of healthy periodontal ligament tissue–derived PDLSCs (H‐PDLSCs, D) and inflammatory periodontal ligament tissue–derived PDLSCs (I‐PDLSCS, E) by the naked eye (left) and under an inverted microscope (right); F, The numbers of single‐cell colonies of different periodontal ligament tissue–derived PDLSCs. N = 3 for each group; *P < .05; G, H, representative flow cytometric profiles show the expression levels of indicated surface markers on H‐PDLSCs (G) and I‐PDLSCs (H); (I‐N) representative images show alizarin red staining of H‐PDLSCs (J) and I‐PDLSCs (M) after osteogenic differentiation induction for 28 d, and oil O red staining of H‐PDLSCs (K) and I‐PDLSCs (N) after adipogenic differentiation induction for 28 d. Blank controls were H‐PDLSCs (I) and I‐PDLSCs (L) without staining. Images on top indicate the images with naked eyes, while those on the bottom indicate photographs acquired with an inverted microscope. Scale bar: A‐E, I, J, L, M, 1 mm; K, N, 100 µm

After culturing the primary periodontal ligament tissues for 3‐5 days, fibroblast‐like cells were found on the edge of the seeded tissues (Figure 1A). Single‐cell clones (Figure 1B), labelled as the first passage of PDLSCs, were selected and expanded by further culturing. PDLSCs in the third to fifth passage (Figure 1C) were used for further experiments.

Both H‐PDLSCs (Figure 1D) and I‐PDLSCs (Figure 1E) formed colonies. I‐PDLSCs formed significantly more colonies (31.0 ± 8.7%) than H‐PDLSCs (25.8 ± 1.4%) (*P* < .05, Figure 1F). As shown in Figure 1G‐H, both H‐PDLSCs and I‐PDLSCs expressed mesenchymal cell markers (Stro‐1, CD146, CD105, CD29 and CD166), but were negative for other markers.

After 28 days of osteogenic differentiation induction, both H‐PDLSCs (Figure 1J) and I‐PDLSCs (Figure 1M) formed mineralized nodules. However, the alizarin red staining was weaker in I‐PDLSCs. After 28 days of adipogenic differentiation induction, both H‐PDLSCs (Figure 1K) and I‐PDLSCs (Figure 1N) exhibited positive oil red staining, and cluster distributed transparent lipid droplets were observed.

### 3.2 Effects of inflammation and nicotine on hPDLSC osteogenic differentiation and inducing osteoclast formation

**Figure 2:**
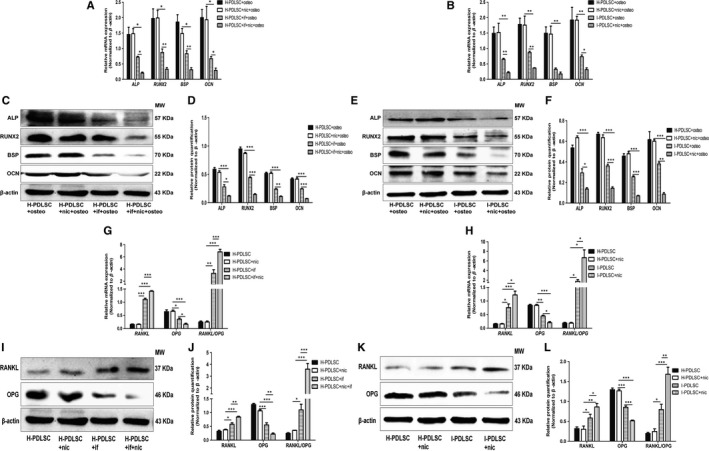
Inflammation and nicotine had synergistic effects on osteogenic differentiation and osteoclast formation‐induction of hPDLSCs. A‐F, In response to osteogenic differentiation induction (osteo), H‐PDLSCs were treated with inflammatory factors alone (if; 5 ng/mL IL‐1β and 10 ng/mL TNF‐α), or nicotine alone (nic; 10^−9^ mol L^−1^), or in combination (if + nic), while I‐PDLSCs were treated with or without nicotine. At 14 d after treatment, mRNA levels of osteogenic differentiation‐associated genes, including *ALP*, *RUNX2*, *BSP* and *OCN*, in H‐PDLSCs (A) and I‐PDLSCs (B), were quantitative by RT‐qPCR. Protein levels of these genes were measured by Western blot. Representative images show targeted proteins in H‐PDLSCs (C) and I‐PDLSCs (E), while the relative quantitation of band intensity is summarized in d‐f. G‐L, H‐PDLSCs were treated with inflammatory factors alone (if; 5 ng/mL IL‐1β and 10 ng/mL TNF‐α), or nicotine alone (nic; 10^−9^ mol L^−1^), or in combination (if + nic), while I‐PDLSCs were treated with or without nicotine. At 14 d after treatment, mRNA levels of osteoclast forming‐related genes *RANKL* and *OPG* in H‐PDLSCs (G) and I‐PDLSCs (H) were quantitated by RT‐qPCR. Representative images show Western blot bands of targeted proteins in H‐PDLSCs (I) and I‐PDLSCs (K), while the relative quantitation of band intensity is summarized in J‐L. N = 3 for each group; *P < .05, **P < .01, ***P < .001. if: inflammatory factors; nic: nicotine; osteo: osteogenic differentiation induction

In response to induction of osteogenic differentiation, 10^−9^ mol/L nicotine did not statistically impact mRNA and protein expression of *ALP*, *RUNX2*, *BSP* and *OCN* (*P* > .05, Figure 2A,C‐D). Remarkably, upon treatment with inflammatory factors IL‐1β and TNF‐α, expression of these genes significantly decreased in the H‐PDLSCs (*P* < .05, Figure 2A,C‐D). Notably, in H‐PDLSCs treated with inflammatory factors and nicotine, these osteogenic differentiation indicators further decreased (*P* < .05, Figure 2A,C‐D). In addition, compared with H‐PDLSCs, I‐PDLSCs had significantly lower expression of *ALP*, *RUNX2*, *BSP* and *OCN* (*P* < .05, Figure 2B,E,F). In the osteogenic differentiation environment, treatment with nicotine further down‐regulated the mRNA expression of *ALP*, *RUNX2* and *OCN* in I‐PDLSCs (*P* < .05, Figure 2B). Although nicotine did not synergistically decrease mRNA expression of *BSP* in I‐PDLSCs (*P* > .05, Figure 2B), it significantly down‐regulated BSP protein expression and other osteogenic differentiation‐associated genes (*P* < .05, Figure 2E,F).

Similarly, singular treatment with nicotine had no obvious effects on expression of *RANKL* and *OPG* in H‐PDLSCs, while additional treatment with nicotine in inflammatory environment further up‐regulated *RANKL* expression and down‐regulated *OPG* expression (*P* < .05, Figure 2G*,*I,J). Compared with H‐PDLSCs, I‐PDLSCs had significantly up‐regulated expression of *RANKL* and down‐regulated expression of *OPG* (*P* < .05, Figure 2H*,*K,L). Treatment with nicotine in I‐PDLSCs further augmented these expression changes (*P* < .05, Figure 2H*,*K,L). Moreover, the synergistic effects of inflammation and nicotine on the ability of hPDLSCs to regulate osteoclastogenesis were also reflected by the increased ratios of *RANKL*/*OPG* expression (Figure 2G,H*,*J‐L).

### 3.3 Periodontal ligament tissues and hPDLSCs derived from the inflammatory microenvironment have higher α7 nAChR expression

**Figure 3:**
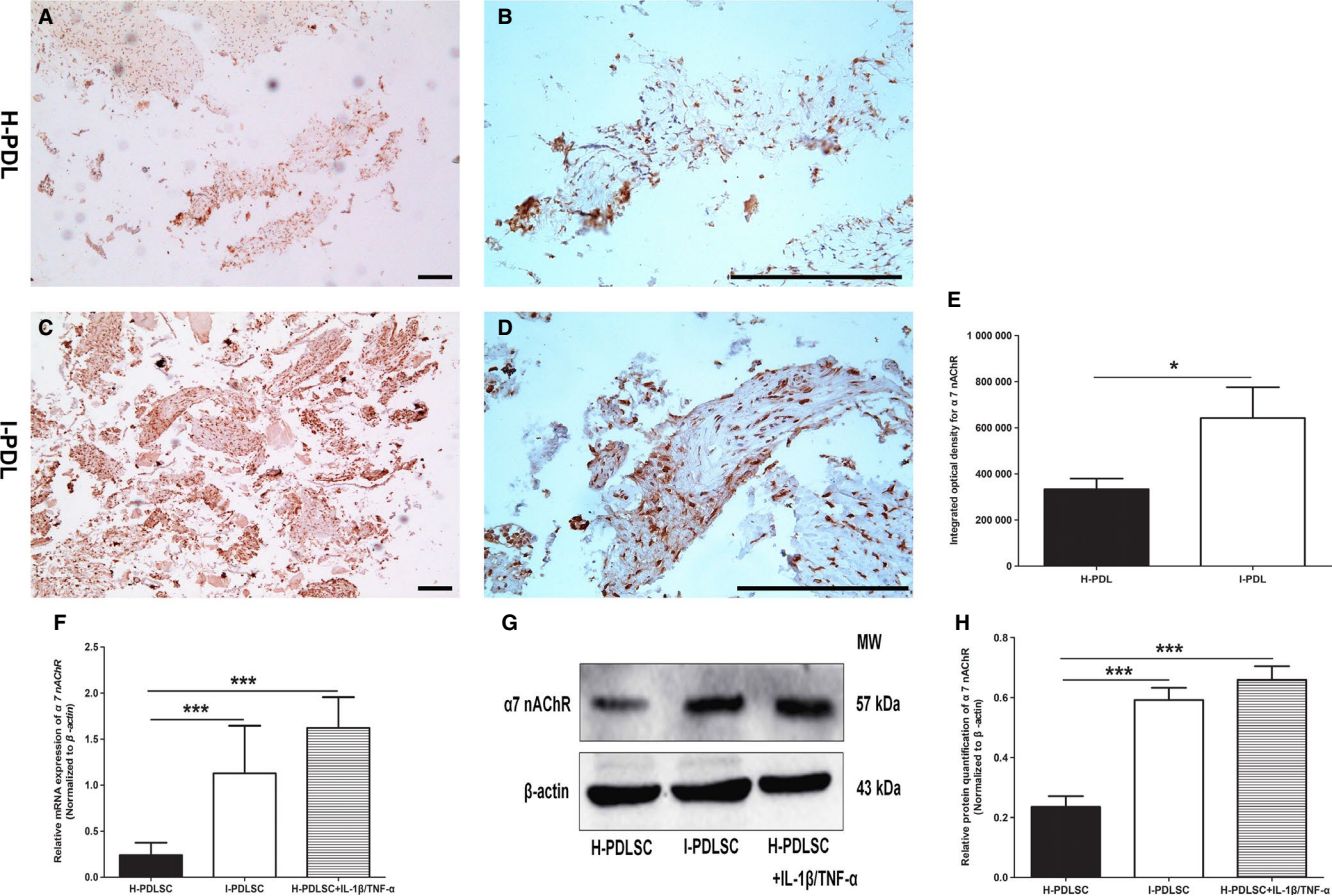
Periodontal ligament tissues and hPDLSCs derived from inflammatory microenvironment had higher expression of α7 nAChR. A‐D, Representative images of immunohistochemical staining of α7 nAChR in periodontal ligament tissues from the healthy (H‐PDL, A, B) and inflamed (I‐PDL, C, D) tissues. Scale bar: 1mm. (E) Semi‐quantitative analysis of immunohistochemical staining of α7 nAChR in H‐PDL and I‐PDL. F, mRNA levels of *α7 nAChR* in H‐PDLSCs, I‐PDLSCs and inflammatory factor–stimulated H‐PDLSC. G, H, Representative Western blot images show the targeted bands in H‐PDLSCs, I‐PDLSCs and inflammatory factor‐stimulated H‐PDLSC (G), and the relative quantitation of band intensity is summarized (H). N = 3 for each group; *P < .05; ***P < .001

As shown in Figure 3A‐D, both periodontal ligament tissues derived from the healthy (H‐PDL) and inflammatory (I‐PDL) environments expressed α7 nAChR. However, I‐PDL had significantly increased α7 nAChR expression (*P* < .05, Figure 3E). Compared with H‐PDLSCs, I‐PDLSCs and IL‐1β/TNF‐α‐treated H‐PDLSCs had significantly increased mRNA (Figure 3F) and protein (Figure 3G,H) expression of α7 nAChR (*P* < .05).

### 3.4 α7 nAChR knock‐down or antagonizing alone does not affect the osteogenic differentiation and osteoclast formation‐induction ability of hPDLSCs in response to inflammation

As shown in Figure S1A,B, qPCR indicated that the silencing efficiency of *α7 nAChR* was 56.6% and 73.0% in H‐PDLSCs and I‐PDLSCs while the α7 nAChR protein expression was reduced by 56.8% and 76.5% in H‐PDLSCs and I‐PDLSCs, respectively (Figure S1C,D).

**Figure 4:**
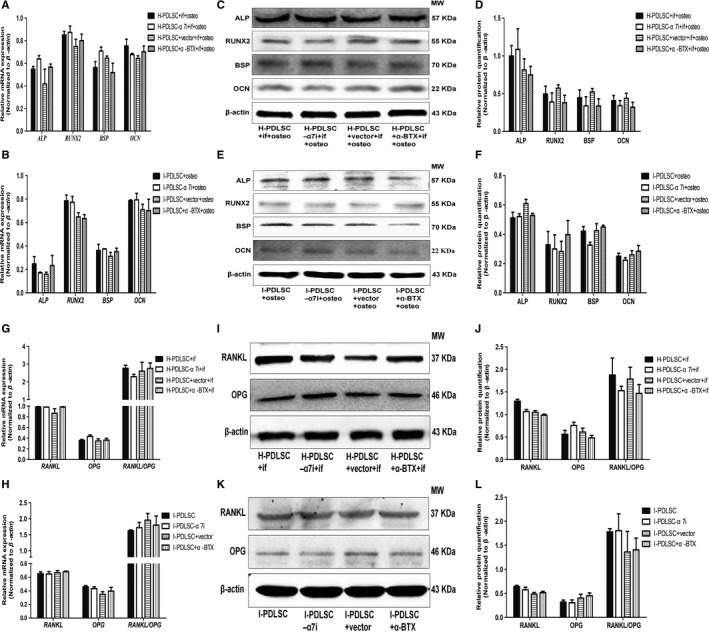
α7 nAChR knock‐down or antagonist alone did not affect the osteogenic differentiation and osteoclast formation of hPDLSCs. A‐F, In response to osteogenic differentiation induction (osteo), uninfected H‐PDLSCs and H‐PDLSCs infected with control shRNA lentivirus (vector) or *α7 nAChR*‐specific shRNA lentivirus (α7i) were treated with inflammatory factors (if; 5 ng/mL IL‐1β and 10 ng/mL TNF‐α), and the specific α7 nAChR antagonist, α‐BTX, or left untreated; I‐PDLSCs were infected with control shRNA lentivirus (vector) or *α7 nAChR*‐specific shRNA lentivirus (α7i) and were treated with α‐BTX or left untreated. At 14 d after treatment, cells were harvested to measure mRNA and protein levels of osteogenic differentiation‐associated genes by RT‐qPCR (A, B) and Western blot (C‐F). Bar graphs show the relative quantitation of mRNA expression in H‐PDLSCs (A) and I‐PDLSCs (B), and protein expression in H‐PDLSCs (D) and I‐PDLSCs (F), and images show the representative results of Western blot in H‐PDLSCs (C) and I‐PDLSCs (E). G‐L, H‐PDLSCs and I‐PDLSCS were treated as in A‐F, except that cells were not induced with osteogenic differentiation condition. Cells were harvested to measure mRNA and protein expression of osteoclast formation‐associated genes *RANKL* and *OPG* by RT‐qPCR (G, H) and Western blot (I‐L). N = 3 for each group; α‐BTX: α‐bungarotoxin; α7i: silencing the expression of α7 nAChR; if: inflammatory factors; osteo: osteogenic differentiation induction

In response to osteogenic differentiation induction, α7 nAChR knock‐down did not significantly alter ALP, RUNX2, BSP and OCN expression (*P* > .05, Figure 4A,C,D). Furthermore, treatment with α‐BTX, a specific α7 nAChR antagonist, also did not significantly impact mRNA and protein expression of the osteogenic differentiation markers (Figure 4A,C,D). Similarly, α7 nAChR inhibition in I‐PDLSCs following osteogenic differentiation induction did not change expression of the osteogenic differentiation‐associated markers (Figure 4B,E,F). Moreover, neither α7 nAChR knock‐down nor the antagonist significantly alter the expression of *RANKL* and *OPG* in either H‐PDLSCs (Figure 4G,I,J) or I‐PDLSCs (Figure 4H,K,L).

### 3.5 Silencing of α7 nAChR abrogates nicotine‐induced impairment on osteogenic differentiation and enhancement on osteoclast formation‐induction of hPDLSCs in the inflammatory microenvironment

**Figure 5:**
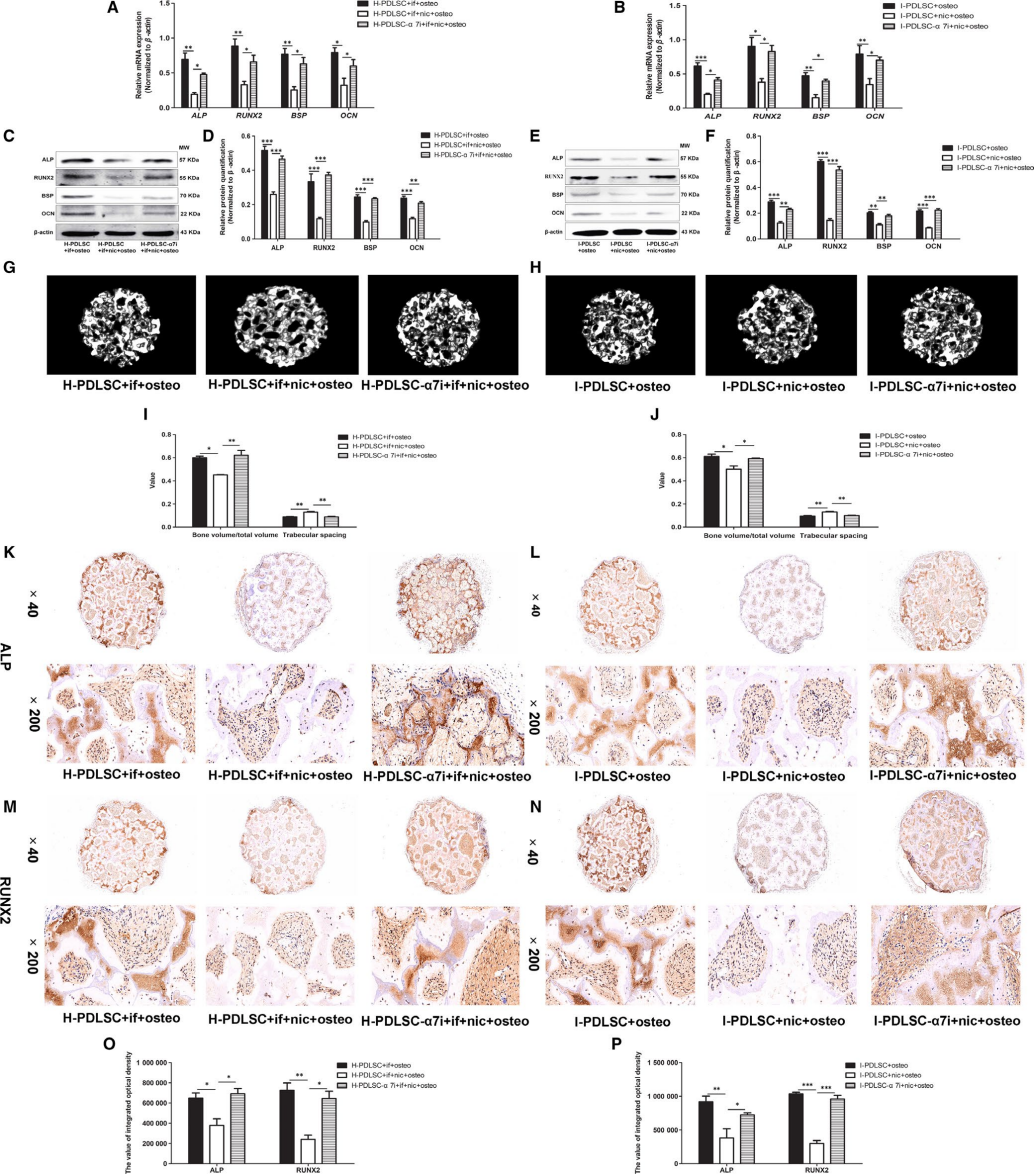
α7 nAChR knock‐down abrogated nicotine‐induced impairment of hPDLSC osteogenic differentiation in the inflammatory microenvironment. A‐F, H‐PDLSCs and I‐PDLSCs were treated as described in Figure 4A‐F, except cells were additionally treated with 10^−9^ mol L^−1^ nicotine (nic). Cells were harvested to measure mRNA and protein levels of osteogenic differentiation‐associated genes by RT‐qPCR (A, B) and Western blot (C‐F). G‐J, Ceramic hydroxyapatite coated with differently treated H‐PDLSCs (G) and I‐PDLSCs (H) was subcutaneously implanted into the dorsal region of nude mice. At 8 wk after implantation, the ceramic hydroxyapatite was subjected to micro‐CT analysis to examine structural changes. Quantitative analysis was conducted to determine bone volume/total volume and trabecular spacing (I, J). K‐P, Changes of protein expression of ALP (K, L) and RUNX2 (M, N) on ceramic hydroxyapatite coated with differently treated H‐PDLSCs (K, M) and I‐PDLSCs (L, N) were determined by immunohistochemical staining. Bar graphs indicate semi‐quantitative analysis of staining for ALP and RUNX2 in H‐PDLSCs (O) and I‐PDLSCs (P). N = 3 for each group; *P < .05, **P < .01, ***P < .001; α7i, silencing the expression of α7 nAChR; if: inflammatory factors; nic: nicotine; osteo, osteogenic differentiation induction

The synergistic effect of nicotine and inflammation was demonstrated by significantly decreased expression of osteogenic differentiation indicator genes in H‐PDLSCs, and this effect was reversed after silencing α7 nAChR expression (*P* < .05, Figure 5A,C,D). Similarly, studies in I‐PDLSCs showed that the synergistic effect of nicotine and inflammation could be significantly abrogated by silencing α7 nAChR expression (*P* < .05, Figure 5B,E,F).

Micro‐CT scanning confirmed that inflammation and nicotine administration synergistically suppressed the formation of osseous hard tissues (Figure 5G,H). This effect was partially reversed by suppressing α7 nAChR expression, which was further supported by quantitative analysis of bone volume vs total volume and trabecular spacing (*P* < .05, Figure 5I,J). Moreover, immunohistochemical staining showed that the synergistic effect of nicotine and inflammation on suppressing ALP (Figure 5K,L) and RUNX2 (Figure 5M,N) expression was partially reversed by down‐regulating α7 nAChR expression. This was further supported by semi‐quantitative analysis between the control hPDLSCs and *α7 nAChR*‐specific shRNA virus‐infected hPDLSCs (*P* < .05, Figure 5O,P).

**Figure 6:**
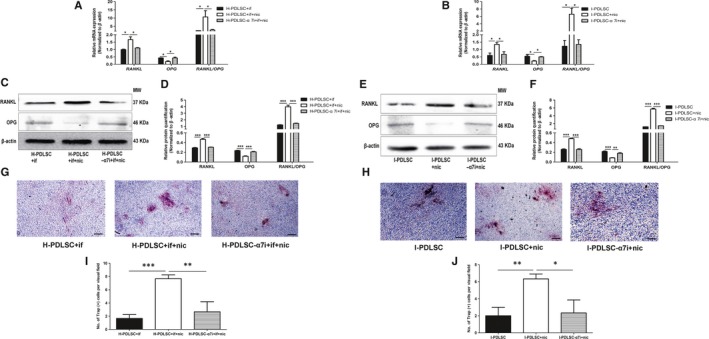
α7 nAChR knock‐down abrogated nicotine‐induced enhancement on osteoclast formation‐induction of hPDLSCs in the inflammatory microenvironment. A‐F, H‐PDLSCs and I‐PDLSCs were treated as described in Figure 4G‐l, except cells were additionally treated with 10^−9^ mol L^−1^ nicotine (nic). mRNA and protein expression of osteoclast formation‐associated genes *RANKL* and *OPG* were quantitated by RT‐qPCR (A, B) and Western blot (C‐F). Bar graphs show relative quantitation of mRNA expression in H‐PDLSCs (A) and I‐PDLSCs (B), and protein expression in H‐PDLSCs (D) and I‐PDLSCs (F), and images show the representative results of Western blot in H‐PDLSCs (C) and I‐PDLSCs (E). G‐J, After co‐culturing with RAW264.7 cells, H‐PDLSCs (G) and I‐PDLSCs (H) with indicated treatments were subjected to TRAP staining to measure the number of induced multinucleate cells. The number of TRAP‐positive cells per visual field was also calculated (I, J). N = 3 for each group; *P < .05, **P < .01, ***P < .001; scale bar: 0.5 mm; α7i: silencing the expression of α7 nAChR; if: inflammatory factors; nic: nicotine

Compared with inflammation alone, additional administration of nicotine further up‐regulated *RANKL* and down‐regulated *OPG* expression in H‐PDLSCs (*P* < .05, Figure 6A). The synergistic effect of nicotine was attenuated by silencing α7 nAChR expression (Figure 6A). Western blot assays showed similar trends for protein expression of these markers in H‐PDLSCs (*P* < .05, Figure 6C,D). In addition, knock‐down of α7 nAChR in I‐PDLSCs also abrogated the effect of nicotine on regulating mRNA (Figure 6B) and protein (Figure 6E,F) expression of *RANKL* and *OPG*.

As shown in Figure 6G,H, inflammation and nicotine synergistically promoted formation of TRAP^+^ multinuclear osteoclast precursors in both H‐PDLSCs and I‐PDLSCs. Importantly, this effect was partially reversed by down‐regulating α7 nAChR expression, which was further supported by comparing the numbers of TRAP^+^ cells per visual field (*P* < .05, Figure 6I,J).

### 3.6 Inflammation‐induced up‐regulated α7 nAChR expression in hPDLSCs is partially dependent on phosphorylated GSK‐3β

**Figure 7:**
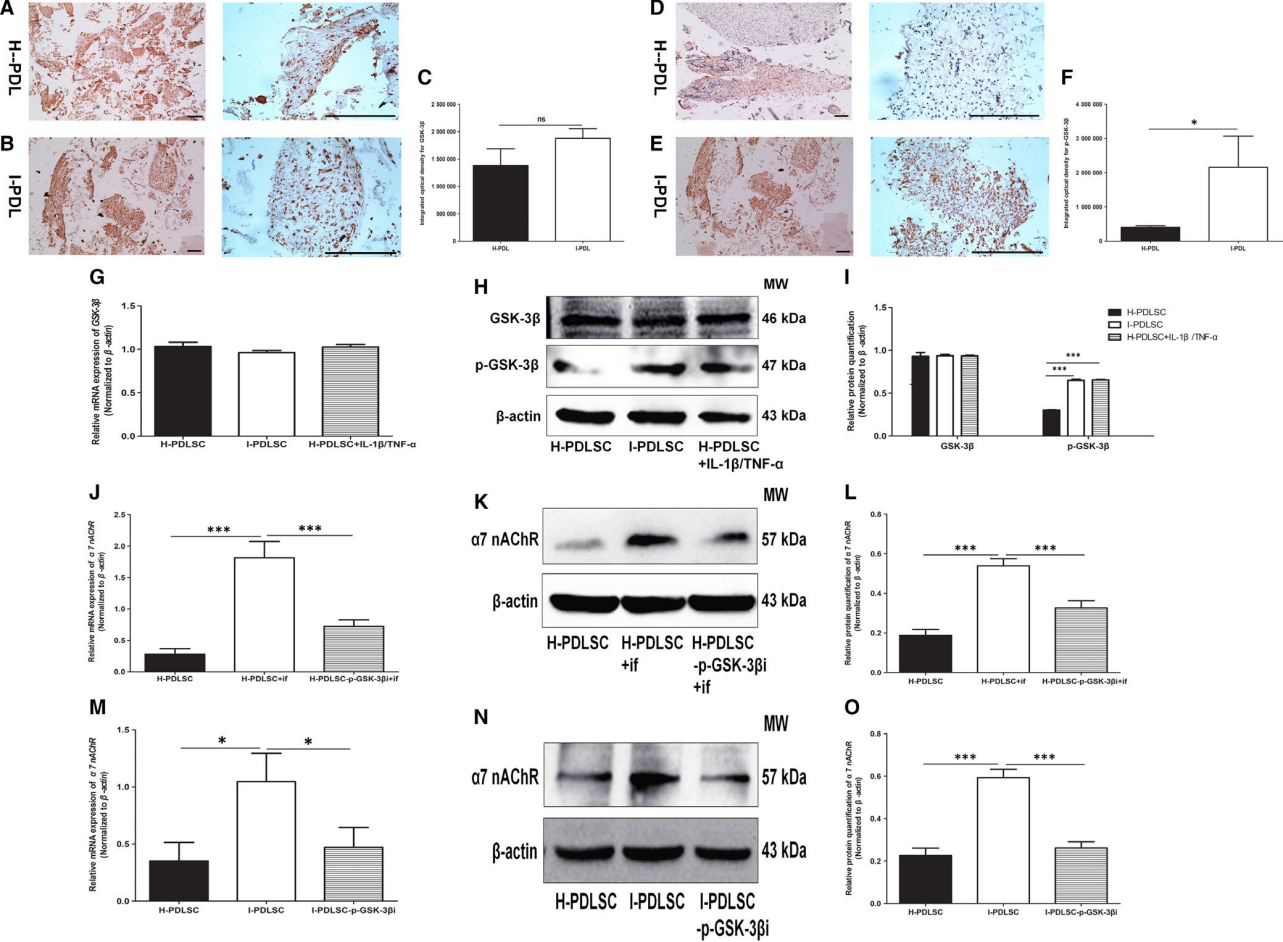
Phosphorylated GSK‐3β up‐regulated α7 nAChR expression in hPDLSCs. A‐F, Expression levels of GSK‐3β (A‐C) and phosphorylated GSK‐3β (p‐GSK‐3β) (D‐F) in H‐PDL (A, D) and I‐PDL (B, E) were determined by immunohistochemical staining. Semi‐quantitative analysis of GSK‐3β expression (C) and p‐GSK‐3β expression (F) is shown. G‐I, mRNA and protein expression of GSK‐3β and p‐GSK‐3β in H‐PDLSCs, I‐PDLSCs and inflammatory factor–stimulated H‐PDLSCs were determined by RT‐qPCR (G) and Western blot (H, I), respectively. (J‐O) Knock‐down of GSK‐3β expression in inflammatory factors stimulated H‐PDLSCs (J‐L) and I‐PDLSC (M‐O) decreased α7 nAChR expression. mRNA levels of *α7 nAChR* were determined by RT‐qPCR (J, M), and protein expression was determined by Western blot (K, L, N, O). N = 3 for each group; *P < .05; ***P < .001; scale bar: 1 mm; if: inflammatory factors; p‐GSK‐3β: phosphorylated GSK‐3β; p‐GSK‐3βi: silencing of p‐GSK‐3β through shRNA lentivirus infection

As shown in Figure 7A,B, both H‐PDL and I‐PDL had comparable expression of GSK‐3β (*P* > .05, Figure 7C). However, the expression level of phosphorylated GSK‐3β (p‐GSK‐3β) in I‐PDL was significantly higher compared to H‐PDL (*P* < .05, Figure 7D‐F). In addition, inflammation did not up‐regulate the transcriptional expression of *GSK‐3β* in hPDLSCs (Figure 7G). However, it significantly regulated the protein levels of p‐GSK‐3β, as I‐PDLSCs and IL‐1β/TNF‐α‐stimulated H‐PDLSCs had more p‐GSK‐3β expression compared to un‐stimulated H‐PDLSCs (Figure 7H,I).

RT‐qPCR analysis confirmed effective silencing of *GSK‐3β* mRNA with an efficiency of 73.3% in H‐PDLSCs (Figure S2A) and 65.2% in I‐PDLSCs (Figure S2B). Western blot analysis showed effective silencing of p‐GSK‐3β at 35.7% and 46.4% in H‐PDLSCs and I‐PDLSCs, respectively (Figure S2C,D).

As shown in Figure 7J,M, knock‐down of GSK‐3β significantly down‐regulated *α7 nAChR* in inflammation‐induced H‐PDLSCs and I‐PDLSCs, both of which had higher transcriptional levels of *α7 nAChR* compared to un‐stimulated H‐PDLSCs previously. Similarly, inflammation‐induced up‐regulation of α7 nAChR protein expression was also abrogated by shRNA‐mediated silencing of *GSK‐3β* in IL‐1β/TNF‐α‐stimulated H‐PDLSCs (Figure 7K,L) and I‐PDLSCs (Figure 7N,O).

## 4 DISCUSSION

In this study, we isolated PDLSCs from healthy and inflamed periodontal ligament tissues. Our systemic characterization indicated that nicotine and inflammation synergistically reduced the osteogenic differentiation capability of PDLSCs, while increasing their capability of osteoclast formation‐induction. Inflammation up‐regulated α7 nAChR expression in both periodontal ligament tissues and PDLSCs, which significantly contributed to the synergistic effect of nicotine and inflammation in periodontitis. We further identified that increased α7 nAChR expression in hPDLSCs in response to inflammation was partially dependent on levels of phosphorylated GSK‐3β.

As PDLSCs are favourable seed cells for periodontal regeneration in tissue engineering, many studies have recently investigated their biological characteristics and regulatory factors. Previous reports indicated that ageing significantly affects the biological behaviour of PDLSCs, as PDLSCs from senior donors have decreased multi‐lineage differentiation capability in vitro 23 and less osseous tissues formed in vivo.^24^ Thus, adequate attention should be paid to the effect of ageing on PDLSCs in clinical and basic research.^25^ Here, we focused on the effect of inflammation on α7 nAChR expression and function in PDLSCs. It is well known that I‐PDLSCs are mainly isolated from periodontitis teeth from senior patients. Wu et al found that matrix secretion and regenerative function of PDLSC cell sheets did not differ between donors younger than 30 years old. Only donors older than 45 years displayed decreased biological behaviour in their PDLSCs.^26^ To overcome the effect of ageing in our study, we recruited patients with an average age of 33.7 years, with the oldest being 35 years old. Therefore, our study design effectively excluded the effect of ageing on osteogenic differentiation and osteoclast formation in both H‐PDLSCs and I‐PDLSCs.

Inflammation can affect the biological characteristics of PDLSCs. Yang et al^27^ found that inflammatory factors involved in periodontitis development, such as TNF‐α and IL‐1β, increased the proliferation ability of PDLSCs in vitro, which is consistent with the findings in this study. As a result, some scholars suggested the application of inflammatory factors at a lower concentration to stimulate proliferation of mesenchymal stem cells in tissue engineering regeneration therapy.^28^ However, inflammation can be a double‐edged sword in PDLSCs. The multipotent differentiation capability of PDLSCs can deteriorate as the concentration of inflammatory factors increases, which leads to an unsatisfactory result of periodontal clinical regeneration.^29^

Clinical observations have indicated that periodontitis patients who smoke consistently have more severe hard tissue resorption.^10^ Under inflammatory conditions, the toxic effect of smoking was not due to a simple accumulation. Smoking, which alone does not lead to periodontal tissue resorption, can exacerbate periodontitis symptoms in response to local inflammation.^30^ This clinical phenomenon suggests that toxic substances in cigarettes synergistically work to increase periodontitis. Our study demonstrated that the in vitro synergistic effect of nicotine and inflammation on osteogenic differentiation and osteoclast formation of PDLSCs was more potent than either stimulation with nicotine or inflammatory factors alone (Figure 2). This finding was in accordance with previous clinical observations.

Our previous work indicated that low concentrations of nicotine (10^−9^‐10^−12^ mol/L) had significantly decreased toxicity to human periodontal ligament cells.^14^ Based on this, we chose to use nicotine at a concentration of 10^−9^ mol/L in this study. Our results confirmed that nicotine at 10^−9^ mol/L did not affect the osteoblastogenesis/osteoclastogenesis balance of PDLSCs, but significantly exacerbated the destructive effect of inflammatory factors (Figure 2). To the best of our knowledge, this is the first report to demonstrate direct evidence of the in vitro synergistic effect of nicotine and inflammation in human PDLSCs.

α7 nAChR is a classical receptor that plays a role in the physiological regulation of neurons.^31^ After identifying its expression in oral tissues,^32^ α7 nAChR was considered a critical molecular regulatory target of oral diseases.^14^ This study further suggests that α7 nAChR expression levels varied in periodontal tissues derived from different microenvironments (Figure 3). These results are consistent with an experimental periodontitis animal model,^12^ which also suggests an important role of α7 nAChR in periodontal inflammatory diseases.

In addition, the finding that inflammation up‐regulates α7 nAChR expression is supported by results obtained in macrophages.^33^ However, contradictory conclusions were reached from other investigations that focused on α7 nAChR expression in the nervous system. Inflammation in the central nervous system down‐regulated α7 nAChR expression, which led to amyloid deposition and further mimicked the phenotype of central nervous degenerative diseases.^34^ However, no clear mechanisms were clarified focusing on the different phenomena.^35^ As a result, the mechanism by which inflammation up‐regulates α7 nAChR expression in periodontal tissues and stem cells requires further investigation.

After confirming that nicotine and inflammation synergistically affected the balance of osteoblastogenesis and osteoclastogenesis in PDLSCs, we knocked down α7 nAChR expression in PDLSCs to further investigate the effects of loss of function. We showed that α7 nAChR knock‐down partially reversed the synergistic effect of nicotine and inflammation (Figures 6 and 7). This finding further expands our understanding about the pathological mechanisms of smoking‐related periodontitis. Considering the findings in the previous research,^12^ α7 nAChR might play a more critical role in regulating the local microenvironment in periodontal tissues. That is, inflammatory factors up‐regulate α7 nAChR expression in periodontal tissues. Nicotine binding to α7 nAChR increased release of inflammatory factors. Thus, a positive feedback loop initiated by local inflammation can result in the imbalance of osteoblastogenesis and osteoclastogenesis of PDLSCs, exacerbating periodontitis in patients who smoke.

GSK‐3β actively participates in inflammatory responses. It has already been demonstrated that inflammatory responses induced by lipopolysaccharides can suppress GSK‐3β activity to regulate expression of inflammatory factors.^36^ Functionally, suppressing GSK‐3β can also regulate the nuclear factor‐κB signalling pathway to affect inflammatory responses of monocytes and macrophages.^37^ Our study also confirmed that in response to inflammation, GSK‐3β activation in periodontal tissues was suppressed, as demonstrated by the up‐regulated expression of phosphorylated GSK‐3β (Figure 7A‐I).

In an inflammatory environment, phosphorylated GSK‐3β regulates the expression of TNF‐α through the wnt pathway to suppress osteogenic differentiation of PDLSCs.^38^ Here, we further demonstrated that suppressing GSK‐3β activity regulated the expression of α7 nAChR (Figure 7J‐O). Knock‐down of GSK‐3β partially reversed the effect of inflammatory factors on up‐regulating α7 nAChR expression. This finding is in accordance with the results in degenerative diseases in the central nervous system.^19^ Up‐regulated expression of amyloid β can suppress expression of phosphorylated GSK‐3β, leading to decreased expression and diminished function of α7 nAChR.^39^, ^40^ Thus, we speculated that in smoking‐related periodontitis, decreasing phosphorylation of GSK‐3β should be an effective therapy.

In summary, inflammation up‐regulates α7 nAChR expression in human periodontal ligament tissues and PDLSCs. Up‐regulated α7 nAChR expression significantly contributes to the synergistic effect of nicotine and inflammation, which exacerbates tissue destruction in smoking‐related periodontitis patients. Increased α7 nAChR expression in response to inflammation is partially dependent on the phosphorylation of GSK‐3β in PDLSCs. Our study provides an experimental basis for clarifying the synergistic effect of nicotine and inflammation in the pathological development of smoking‐related periodontitis. In future work, detailed molecular mechanisms and research in smoking‐related periodontitis animal models might be explored.

## CONFLICT OF INTEREST

The authors confirm that there are no conflicts of interest.

## AUTHOR CONTRIBUTIONS

XW, YY and ZZ designed the experiments; ZZ, FL, LW and BZ performed the in vitro experiments; FL, YC, and YY performed the animal‐related experiments; FL and XW analysed the data; and ZZ, FL and YY wrote the manuscript.

## Data Availability

The data that support the findings of this study are available from the corresponding author upon reasonable request.
