# Supplementary figures and images for: Inflammation has synergistic effect with nicotine in periodontitis by up‐regulating the expression of α7 nAChR via phosphorylated GSK‐3β
Source: J Cell Mol Med. 2020 Jan 13;24(4):2663–76. doi: 10.1111/jcmm.14986 (PMC7028870; doi:10.1111/jcmm.14986)

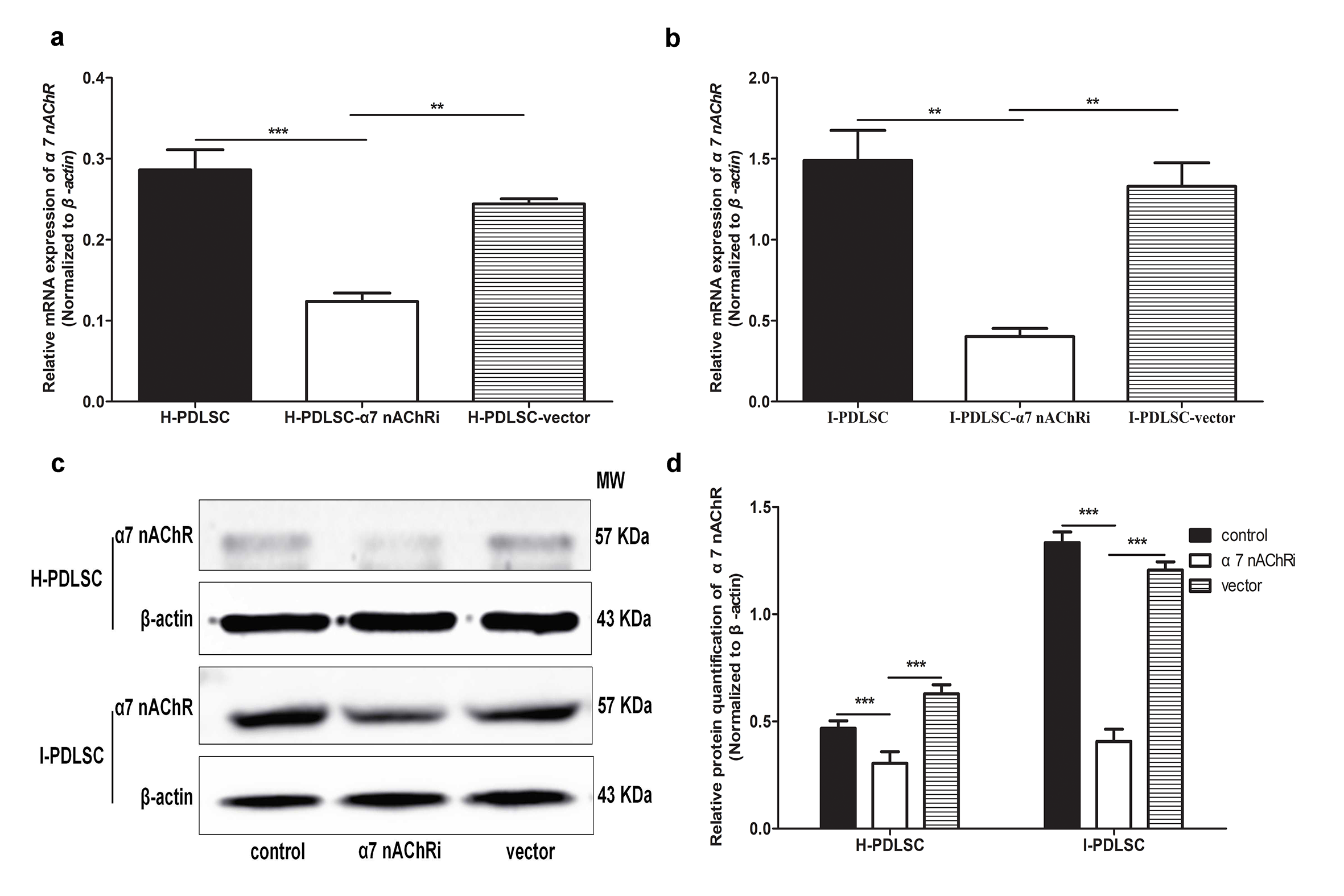

Supplement: Supplementary file 1 [file JCMM-24-2663-s001.tif]

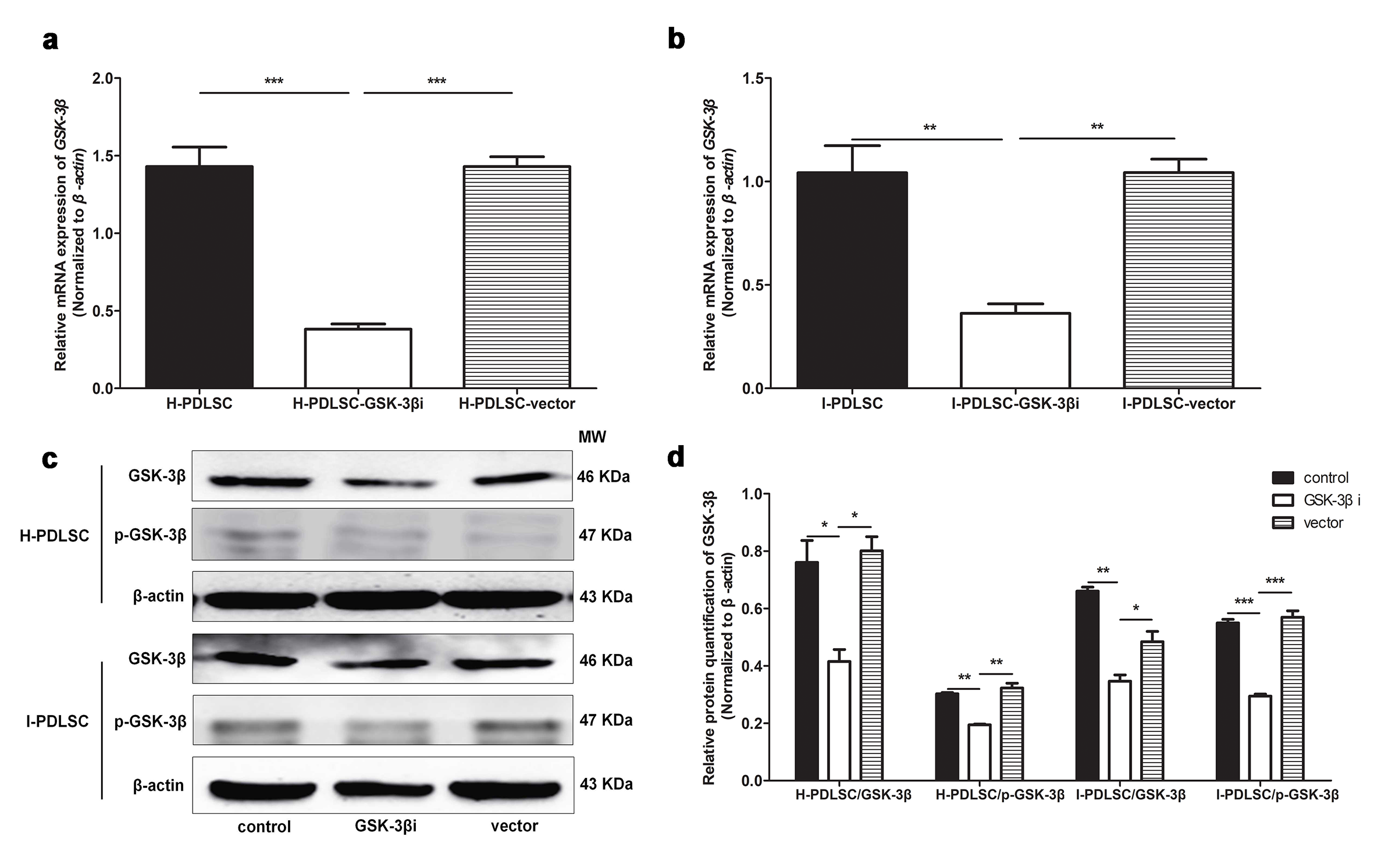

Supplement: Supplementary file 2 [file JCMM-24-2663-s002.tif]
