# Supplementary material for: Inflammation has synergistic effect with nicotine in periodontitis by up‐regulating the expression of α7 nAChR via phosphorylated GSK‐3β
Source: J Cell Mol Med. 2020 Jan 13;24(4):2663–76. doi: 10.1111/jcmm.14986 (PMC7028870; doi:10.1111/jcmm.14986)
Supplement: Supplementary file 3 [file JCMM-24-2663-s003.docx]

**Table S1** **Primer sequences used for RT-qPCR in this study**

| **Gene** | **Primer sequence (5’ – 3’)** | **Accession number** |
| --- | --- | --- |
| *h-ALP-F1234* | CCTTGTAGCCAGGCCCATTG | NM_000478.6 |
| *h-ALP-R* | GGACCATTCCCACGTCTTCAC |  |
| *h-RUNX2-F* | CACTGGCGCTGCAACAAGA | NM_001024630.4 |
| *h-RUNX2-R* | CATTCCGGAGCTCAGCAGAATAA |  |
| *h-BSP-F* | GGGCAGTAGTGACTCATCCGA | NM_004967.4 |
| *h-BSP-R* | TCTTCATTGTTTTCTCCTTCATTTG |  |
| *h-OCN-F* | CCCAGGCGCTACCTGTATCAA | NM_199173.6 |
| *h-OCN-R* | GGTCAGCCAACTCGTCACAGTC |  |
| *h-RANKL-F* | ATCACAGCACATCAGAGCAGAGA | NM_003701.4 |
| *h-RANKL-R* | AGGACAGACTCACTTTATGGGAAC |  |
| *h-OPG-F* | GGAACCCCAGAGCGAAATACA | NM_002546.4 |
| *h-OPG-R* | GGGAACAGCAAACCTGAAGAATG |  |
| *h-CHRNA7-F* | GAGCCCTACCCCGATGTCA | NM_000746.5 |
| *h-CHRNA7-R* | ATCTCAGCCACGAGCAGCA |  |
| *h-GSK3B-F* | TCCCTCAAATTAAGGCACATC | NM_001354596.1 |
| *h-GSK3B-R* | CACGGTCTCCAGTATTAGCATCT |  |
| *h-ACTB-F* | TGGCACCCAGCACAATGAA | NM_001101.5 |
| *h-ACTB-R* | CTAAGTCATAGTCCGCCTAGAAGCA |  |
