# Supplementary material for: Inflammation has synergistic effect with nicotine in periodontitis by up‐regulating the expression of α7 nAChR via phosphorylated GSK‐3β
Source: J Cell Mol Med. 2020 Jan 13;24(4):2663–76. doi: 10.1111/jcmm.14986 (PMC7028870; doi:10.1111/jcmm.14986)
Supplement: Supplementary file 4 [file JCMM-24-2663-s004.docx]

**Figure S1** α7 nAChR shRNA efficiently knocked down α7 nAChR expression in hPDLSCs. (a-d) H-PDLSCs and I-PDLSCs were left untreated or infected with control shRNA or α7 nAChR-specific shRNA lentiviral particles. Cells were harvested at 24 hours after infection. mRNA levels of α7 nAChR in H-PDLSCs (a) and I-PDLSCs (b) were quantitated by RT-qPCR. Representative Western blot images show the bands of targeted proteins in indicated cells (c), and the relative quantitation of band intensity is summarized (d). N = 3 for each group; ** P < 0.01; *** P < 0.001.

**Figure S2** Infection of GSK-3β–specific shRNA lentiviral particles efficiently knocked down p-GSK-3β in hPDLSCs. (a-b) H-PDLSCs and I-PDLSCs were left untreated, or were infected with control shRNA or GSK-3β -specific shRNA lentiviral particles, and cells were harvested at 24 hours after infection. mRNA levels of GSK-3β in H-PDLSCs (a) and I-PDLSCs (b) were quantified by RT-qPCR. (c-d) Representative Western blot images show the bands of targeted proteins in indicated cells (c), and relative band intensity in indicated cells is summarized (d). N = 3 for each group; * P < 0.05, ** P < 0.01.
